# Supplementary material for: Mediators of monocyte chemotaxis and matrix remodeling are associated with mortality and pulmonary fibroproliferation in patients with severe COVID-19
Source: PLoS One. 2024 Aug 6;19(8):e0285638. doi: 10.1371/journal.pone.0285638 (PMC11302896; doi:10.1371/journal.pone.0285638)
Supplement: S1 File — (DOCX) [file pone.0285638.s001.docx]

**Supplemental Data:**

**Supplemental Table 1:**

| **Biomarker Quality Control - Plasma** | | | | | | |
| --- | --- | --- | --- | --- | --- | --- |
| **Biomarker** | **Lower limit of detection (avg, pg/mL)** | **Upper limit of detection (pg/mL)** | **Out of range/imputed (%)** | **Average Intraplate % CV** | **Interplate %CV** | **Included in analysis** |
| IL-6 | .098 | 767 | 1 | 8.71 | 4.65 | Yes |
| IFN-γ | .11 | 1380 | 6 | 8.1 | 4.8 | Yes |
| TNF-α | .028 | 390 | 1 | 5.9 | 4.9 | Yes |
| CCL-2/MCP-1 | 0.02 | 544 | 0 | 5.4 | 10.4 | Yes |
| CCL-13/MCP-4 | 1.72 | 683 | 0 | 6 | 18.9 | Yes |
| Amphiregulin | 1.1 | 2600 | 0 | 7.6 | 23.8 | Yes |
| MMP-9 | 2.3 | 100000 | 0.3 | 4.8 | 15.2 | Yes |
| MMP-7 | 0.67 | 1000 | 0 | 4 | 7.5 | Yes |
| P-Selectin | 5.1 | 200000 | 0 | 3.4 | 7.2 | Yes |
| S100A12 | 15.7 | 50000 | 26 | 10.3 | 26.2 | No |
| SDF-1a/CXCL12 | 3432 | 1020000 | 7.7 | 11.3 | 22.7 | Yes |
| TGF-β1 | 144 | 178500 | 2.5 | 22 | 37.3 | No |

| **Biomarker Quality Control – Endotracheal Aspirate (ETA)** | | | | | | |
| --- | --- | --- | --- | --- | --- | --- |
| **Biomarker** | **Lower limit of detection (avg, pg/mL)** | **Upper limit of detection (pg/mL)** | **Out of range/imputed (%)** | **Average Intraplate % CV** | **Interplate %CV** | **Included in analysis** |
| IL-6 | .04 | 767 | 0 | 20.8 | 48.7 | No |
| IFN-γ | .11 | 138 | 67 | 1.5 | 12.1 | No |
| TNF-α | .03 | 390 | 3.5 | 1.4 | 3.5 | Yes |
| CCL-2/MCP-1 | .06 | 544 | 2.5 | 3.2 | 14.9 | Yes |
| CCL-13/MCP-4 | 1.17 | 683 | 33 | 9.3 | 68.9 | No |
| Amphiregulin | 0.46 | 2600 | 0 | NA | 6.7 | Yes |
| MMP-9 | 1.03 | 100000 | 0 | NA | 4.8 | Yes |
| MMP-7 | 0.95 | 10000 | 3.5 | 2.2 | 5.9 | Yes |
| P-Selectin | 1.8 | 200000 | 1.4 | NA | 12.5 | Yes |
| S100A12 | 50000 | 13.4 | 9.7 | 6.7 | 7.1 | Yes |
| SDF-1a/CXCL12 | 934 | 620000 | 52.6 | NA | 44 | No |
| TGF-β1 | 178500 | 748 | 68.4 | NA | NA | No |

**Quality control metrics of immunoassays performed on plasma and endotracheal aspirates (ETA)**. Analytes that did not meet any of the following quality control parameters were excluded from analysis: 1) intraplate %CV>25%, 2) interplate %CV>25%, or 3) >10% of samples with measurement below the lower limit of detection

CV-- coefficient of variance

**Supplemental Table 2:**

| **Mediator** | **Unadjusted** | | | | **Age, Sex** | | | |
| --- | --- | --- | --- | --- | --- | --- | --- | --- |
|  | **OR** | **CI (2.5%)** | **CI (97.5%)** | **p-value** | **OR** | **CI (2.5%)** | **CI (97.5%)** | **p-value** |
| CCL2 | 1.356 | 1.104 | 1.7 | 0.00541 (*) | 1.404 | 1.124 | 1.797 | 0.0042 (*) |
| MCP-4 | 1.189 | 0.733 | 1.94 | 0.483 | 1.211 | 0.724 | 2.04 | 0.466 |
| IL-6 | 1.26 | 1.115 | 1.45 | 0.00043 (*) | 1.307 | 1.14 | 1.52 | 2.00E-04 (*) |
| TNF-a | 2 | 1.46 | 2.84 | 3.85E-05 (*) | 1.97 | 1.42 | 2.83 | 0.000118 (*) |
| IFN-g | 0.998 | 0.9007 | 1.1 | 0.973 | 1.02 | 0.91 | 1.14 | 0.753 |
| MMP-9 | 1.1 | 0.927 | 1.3 | 0.283 | 1.09 | 0.91 | 1.32 | 0.356 |
| CXCL12 | 1.48 | 1.12 | 2.01 | 0.0085 (*) | 1.42 | 1.06 | 1.96 | 0.0229 (*) |
| Amphiregulin | 1.94 | 1.42 | 2.78 | 1.09E-04 (*) | 1.85 | 1.33 | 2.7 | 0.00066 (*) |
| MMP-7 | 1.26 | 0.945 | 1.68 | 0.1194 | 1.188 | 0.88 | 1.62 | 0.27 |
| P-selectin | 1.22 | 0.874 | 1.72 | 0.244 | 1.218 | 0.85 | 1.76 | 0.285 |

| **Mediator** | **Age, Sex, Steroids** | | | | **Age, Sex, Steroids, APACHE III Score** | | | |
| --- | --- | --- | --- | --- | --- | --- | --- | --- |
|  | **OR** | **CI (2.5%)** | **CI (97.5%)** | **p-value** | **OR** | **CI (2.5%)** | **CI (97.5%)** | **p-value** |
| CCL2 | 1.51 | 1.194 | 1.96 | 0.000976 (*) | 1.31 | 1.016 | 1.73 | 0.0422 (*) |
| MCP-4 | 1.33 | 0.778 | 2.29 | 0.301 | 1.21 | 0.692 | 2.13 | 0.508 |
| IL-6 | 1.39 | 1.21 | 1.64 | 1.82E-05 (*) | 1.31 | 1.12 | 1.56 | 0.000962 (*) |
| TNF-a | 2.04 | 1.45 | 2.98 | 1.04E-04 (*) | 1.75 | 1.24 | 2.59 | 0.0029 (*) |
| IFN-g | 1.02 | 0.912 | 1.15 | 0.715 | 1.02 | 0.91 | 1.16 | 0.67 |
| MMP-9 | 1.08 | 0.899 | 1.32 | 0.41 | 0.97 | 0.8 | 1.19 | 0.79 |
| CXCL12 | 1.52 | 1.12 | 2.14 | 0.0099 (*) | 1.3 | 0.95 | 1.85 | 0.112 |
| Amphiregulin | 1.9 | 1.36 | 2.78 | 0.0004 (*) | 1.6 | 1.1 | 2.4 | 0.0189 (*) |
| MMP-7 | 1.18 | 0.87 | 1.63 | 0.28 | 1.01 | 0.72 | 1.4 | 0.96 |
| P-selectin | 1.22 | 0.849 | 1.78 | 0.28 | 0.88 | 0.574 | 1.34 | 0.56 |

**Plasma proteins measured at 24h and their association with in-hospital mortality**. Odds ratio (OR), 95% Confidence Interval (CI), and *p*-value for association between log_2_-transformed protein concentrations and in-hospital mortality. Logistic model is unadjusted, or includes age, sex, treatment with steroids, and enrollment APACHE III score as covariates. (*) indicates *p*-value<0.05

**Supplemental Table 3:**

| **Mediator** | **Unadjusted** | | | | **Age, Sex** | | | |
| --- | --- | --- | --- | --- | --- | --- | --- | --- |
|  | **OR** | **CI (2.5%)** | **CI (97.5%)** | **p-value** | **OR** | **CI (2.5%)** | **CI (97.5%)** | **p-value** |
| CCL2 | 1.75 | 1.006 | 3.48 | 0.0691 | 2.26 | 1.13 | 5.51 | 0.0346 |
| MCP-4 | 3.4 | 0.915 | 16.1 | 0.0838 | 3.8 | 0.82 | 22.4 | 0.0989 |
| IL-6 | 1.28 | 0.937 | 1.84 | 0.1415 | 2.1 | 1.2 | 5.1 | 0.0311 |
| TNF-a | 1.75 | 0.894 | 3.87 | 0.121 | 2.455 | 1.07 | 6.55 | 0.0423 |
| IFN-g | 1.26 | 1.11 | 3.24 | 0.0361 (*) | 2.01 | 1.11 | 5.5 | 0.069 |
| MMP-9 | 1.14 | 0.844 | 1.54 | 0.4 | 1.108 | 0.815 | 1.52 | 0.515 |
| CXCL12 | 1.03 | 0.707 | 1.53 | 0.869 | 1.056 | 0.715 | 1.59 | 0.0074 |
| Amphiregulin | 2.09 | 1.44 | 3.15 | 1.98E-04 (*) | 1.95 | 1.33 | 2.96 | 0.00102 |
| MMP-7 | 1.065 | 0.783 | 1.44 | 0.699 | 1.18 | 0.85 | 1.65 | 0.312 |
| P-selectin | 1.27 | 0.759 | 2.16 | 0.369 | 1.39 | 0.8 | 2.45 | 0.244 |

| **Mediator** | **Age, Sex, Steroids** | | | | **Age, Sex, Steroids, APACHE III Score** | | | |
| --- | --- | --- | --- | --- | --- | --- | --- | --- |
|  | **OR** | **CI (2.5%)** | **CI (97.5%)** | **p-value** | **OR** | **CI (2.5%)** | **CI (97.5%)** | **p-value** |
| CCL2 | 2.11 | 1.04 | 5.25 | 0.0612 | 1.97 | 0.94 | 5.02 | 0.1 |
| MCP-4 | 3.1 | 0.65 | 19.04 | 0.17 | 2.62 | 0.43 | 15.7 | 0.25 |
| IL-6 | 2.05 | 1.17 | 5.04 | 0.0417 (*) | 2.03 | 1.14 | 5 | 0.045 (*) |
| TNF-a | 3.42 | 1.29 | 13.83 | 0.0305 (*) | 3 | 0.946 | 15.6 | 0.114 |
| IFN-g | 1.89 | 1.04 | 5.18 | 0.0985 | 1.93 | 1.02 | 6.02 | 0.13 |
| MMP-9 | 1.04 | 0.76 | 1.43 | 0.81 | 1.03 | 0.74 | 1.4 | 0.85 |
| CXCL12 | 1.09 | 0.73 | 1.67 | 0.69 | 1.06 | 0.71 | 1.65 | 0.78 |
| Amphiregulin | 1.8 | 1.21 | 2.77 | 0.0052 (*) | 1.67 | 1.1 | 2.6 | 0.02 (*) |
| MMP-7 | 1.24 | 0.886 | 1.76 | 0.213 | 1.16 | 0.79 | 1.7 | 0.44 |
| P-selectin | 1.31 | 0.74 | 2.3 | 0.36 | 1.2 | 0.66 | 2.17 | 0.56 |

**Plasma proteins measured at 72-168h and their association with in-hospital mortality**. Odds ratio (OR), 95% Confidence Interval (CI), and *p*-value for association between log_2_-transformed protein concentrations and in-hospital mortality. Logistic model is unadjusted, or includes age, sex, treatment with steroids, and enrollment APACHE III score as covariates. (*) indicates *p*-value<0.05

**Supplemental Table 4:**

| **Mediator** | **Unadjusted** | | | | **Age, Sex** | | | |
| --- | --- | --- | --- | --- | --- | --- | --- | --- |
|  | **OR** | **CI (2.5%)** | **CI (97.5%)** | **p-value** | **OR** | **CI (2.5%)** | **CI (97.5%)** | **p-value** |
| CCL2 | 0.51 | 0.36 | 0.37 | 1.72E-05 (*) | 0.51 | 0.36 | 0.69 | 2.97E-05 (*) |
| MCP-4 | 0.75 | 0.45 | 1.23 | 0.266 | 0.74 | 0.446 | 1.22 | 0.25 |
| IL-6 | 0.71 | 0.593 | 0.83 | 6.13E-05 (*) | 0.71 | 0.59 | 0.83 | 8.07E-05 (*) |
| TNF-a | 0.5 | 0.35 | 0.7 | 1.44E-04 (*) | 0.51 | 0.35 | 0.71 | 0.0002 (*) |
| IFN-g | 1.01 | 0.917 | 1.13 | 0.74 | 1.02 | 0.92 | 1.13 | 0.74 |
| MMP-9 | 0.74 | 0.6 | 0.89 | 0.0024 (*) | 0.75 | 0.61 | 0.904 | 0.004 (*) |
| CXCL12 | 0.53 | 0.38 | 0.711 | 5.89E-05 (*) | 0.54 | 0.386 | 0.724 | 1.10E-04 (*) |
| Amphiregulin | 0.43 | 0.28 | 0.61 | 1.40E-05 (*) | 0.42 | 0.27 | 0.6 | 2.40E-05 (*) |
| MMP-7 | 0.57 | 0.4 | 0.79 | 1.16E-03 (*) | 0.586 | 0.41 | 0.81 | 2.00E-03 (*) |
| P-selectin | 0.47 | 0.31 | 0.68 | 1.87E-04 (*) | 0.47 | 0.31 | 0.698 | 2.92E-04 (*) |

| **Mediator** | **Age, Sex, Steroids** | | | | **Age, Sex, Steroids, APACHE III Score** | | | |
| --- | --- | --- | --- | --- | --- | --- | --- | --- |
|  | **OR** | **CI (2.5%)** | **CI (97.5%)** | **p-value** | **OR** | **CI (2.5%)** | **CI (97.5%)** | **p-value** |
| CCL2 | 0.47 | 0.33 | 0.64 | 1.02E-05 (*) | 0.55 | 0.38 | 0.78 | 0.0014 (*) |
| MCP-4 | 0.69 | 0.4 | 1.15 | 0.16 | 0.77 | 0.426 | 1.39 | 0.4 |
| IL-6 | 0.67 | 0.55 | 0.8 | 3.13E-05 (*) | 0.75 | 0.61 | 0.9 | 0.0045 (*) |
| TNF-a | 0.51 | 0.353 | 0.72 | 0.000253 (*) | 0.67 | 0.45 | 0.91 | 0.029 (*) |
| IFN-g | 1.02 | 0.92 | 1.13 | 0.74 | 1.03 | 0.91 | 1.16 | 0.65 |
| MMP-9 | 0.75 | 0.61 | 0.914 | 0.0059 (*) | 0.86 | 0.69 | 1.06 | 0.167 |
| CXCL12 | 0.50 | 0.35 | 0.68 | 4.40E-05 (*) | 0.59 | 0.41 | 0.82 | 0.00266 (*) |
| Amphiregulin | 0.41 | 0.27 | 0.6 | 2.12E-05 (*) | 0.53 | 0.33 | 0.81 | 0.004996 (*) |
| MMP-7 | 0.59 | 0.41 | 0.82 | 2.62E-03 (*) | 0.71 | 0.48 | 1.03 | 0.08 |
| P-selectin | 0.47 | 0.31 | 0.699 | 3.01E-04 (*) | 0.67 | 0.43 | 1.02 | 0.067 |

**Plasma proteins measured at 24h and their association with high Ventilator-Free Days (VFD≥14)**. Odds ratio (OR), 95% Confidence Interval (CI), and *p*-value for association between log_2_-transformed protein concentrations and in-hospital mortality. Logistic model is unadjusted, or includes age, sex, treatment with steroids, and enrollment APACHE III score as covariates. (*) indicates *p*-value<0.05

**Supplemental Table 5:**

| **Mediator** | **Unadjusted** | | | | **Age, Sex** | | | |
| --- | --- | --- | --- | --- | --- | --- | --- | --- |
|  | **OR** | **CI (2.5%)** | **CI (97.5%)** | **p-value** | **OR** | **CI (2.5%)** | **CI (97.5%)** | **p-value** |
| CCL2 | 0.63 | 0.33 | 1.07 | 0.119 | 0.69 | 0.36 | 1.18 | 0.21 |
| MCP-4 | 0.93 | 0.25 | 3.15 | 0.9 | 1.09 | 0.28 | 4.04 | 0.89 |
| IL-6 | 0.64 | 0.392 | 0.913 | 0.033 (*) | 0.63 | 0.377 | 0.93 | 0.0402 (*) |
| TNF-a | 0.51 | 0.201 | 1.05 | 0.102 | 0.56 | 0.227 | 1.16 | 0.16 |
| IFN-g | 0.76 | 0.498 | 1.08 | 0.16 | 0.75 | 0.477 | 1.1 | 0.17 |
| MMP-9 | 0.87 | 0.63 | 1.2 | 0.39 | 0.88 | 0.63 | 1.2 | 0.42 |
| CXCL12 | 0.59 | 0.37 | 0.88 | 0.016 (*) | 0.62 | 0.389 | 0.935 | 0.03 (*) |
| Amphiregulin | 0.66 | 0.44 | 0.95 | 0.03 (*) | 0.61 | 0.404 | 0.91 | 0.0171 (*) |
| MMP-7 | 0.59 | 0.4 | 0.85 | 0.0061 (*) | 0.599 | 0.4 | 0.86 | 0.0083 (*) |
| P-selectin | 0.56 | 0.31 | 0.97 | 0.044 (*) | 0.6 | 0.334 | 1.06 | 0.082 |

| **Mediator** | **Age, Sex, Steroids** | | | | **Age, Sex, Steroids, APACHE III Score** | | | |
| --- | --- | --- | --- | --- | --- | --- | --- | --- |
|  | **OR** | **CI (2.5%)** | **CI (97.5%)** | **p-value** | **OR** | **CI (2.5%)** | **CI (97.5%)** | **p-value** |
| CCL2 | 0.67 | 0.33 | 1.26 | 0.23 | 0.85 | 0.403 | 1.77 | 0.65 |
| MCP-4 | 1.4 | 0.283 | 7.74 | 0.67 | 3.35 | 0.55 | 2.8 | 0.2 |
| IL-6 | 0.63 | 0.350 | 9.76 | 0.068 | 0.64 | 0.33 | 1.04 | 0.11 |
| TNF-a | 0.44 | 0.14 | 0.975 | 0.0734 | 0.68 | 0.19 | 1.95 | 0.5 |
| IFN-g | 0.78 | 0.49 | 1.17 | 0.26 | 0.83 | 0.5 | 1.27 | 0.42 |
| MMP-9 | 0.88 | 0.635 | 1.23 | 0.48 | 0.9 | 0.633 | 1.27 | 0.55 |
| CXCL12 | 0.61 | 0.37 | 0.93 | 0.028 (*) | 0.65 | 0.405 | 1.005 | 0.063 |
| Amphiregulin | 0.64 | 0.41 | 0.97 | 0.04 (*) | 0.67 | 0.43 | 1.04 | 0.08 |
| MMP-7 | 0.57 | 0.38 | 0.83 | 0.00484 (*) | 0.69 | 0.45 | 1.02 | 0.07 |
| P-selectin | 0.64 | 0.35 | 1.12 | 0.123 | 0.65 | 0.344 | 1.17 | 0.158 |

**Plasma proteins measured at 72-168h and their association with high Ventilator-Free Days (VFD≥14)**. Odds ratio (OR), 95% Confidence Interval (CI), and *p*-value for association between log_2_-transformed protein concentrations and in-hospital mortality. Logistic model is unadjusted, or includes age, sex, treatment with steroids, and enrollment APACHE III score as covariates. (*) indicates *p*-value<0.05

**Supplemental Table 6:**

| **Mediator** | **Unadjusted** | | | | **Age, Sex** | | | |
| --- | --- | --- | --- | --- | --- | --- | --- | --- |
|  | **OR** | **CI (2.5%)** | **CI (97.5%)** | **p-value** | **OR** | **CI (2.5%)** | **CI (97.5%)** | **p-value** |
| CCL2 | 0.88 | 0.56 | 1.34 | 0.57 | 0.9 | 0.56 | 1.37 | 0.64 |
| MCP-4 | 1.09 | 0.45 | 2.7 | 0.84 | 1.19 | 0.455 | 3.24 | 0.72 |
| IL-6 | 1.03 | 0.82 | 1.3 | 0.77 | 1.03 | 0.8 | 1.3 | 0.82 |
| TNF-a | 0.814 | 0.47 | 1.32 | 0.42 | 0.84 | 0.48 | 1.38 | 0.5 |
| IFN-g | 0.87 | 0.7 | 1.04 | 0.15 | 0.88 | 0.71 | 1.1 | 0.19 |
| MMP-9 | 1.13 | 0.86 | 1.55 | 0.4 | 1.14 | 0.86 | 1.59 | 0.38 |
| CXCL12 | 1.84 | 0.925 | 4.44 | 0.125 | 1.99 | 0.99 | 4.9 | 0.087 |
| Amphiregulin | 1.4 | 0.77 | 2.77 | 0.26 | 1.7 | 0.88 | 3.59 | 0.12 |
| MMP-7 | 1.18 | 0.64 | 2.24 | 0.58 | 1.17 | 0.62 | 2.2 | 0.63 |
| P-selectin | 1.08 | 0.58 | 2.03 | 0.81 | 1.08 | 0.57 | 2.1 | 0.79 |

| **Mediator** | **Age, Sex, Steroids** | | | | **Age, Sex, Steroids, APACHE III Score** | | | |
| --- | --- | --- | --- | --- | --- | --- | --- | --- |
|  | **OR** | **CI (2.5%)** | **CI (97.5%)** | **p-value** | **OR** | **CI (2.5%)** | **CI (97.5%)** | **p-value** |
| CCL2 | 0.9 | 0.57 | 1.37 | 0.64 | 0.83 | 0.51 | 1.28 | 0.43 |
| MCP-4 | 1.18 | 0.45 | 3.2 | 0.7 | 1.19 | 0.45 | 3.2 | 0.72 |
| IL-6 | 1.03 | 0.8 | 1.3 | 0.81 | 0.98 | 0.76 | 1.26 | 0.88 |
| TNF-a | 0.8 | 0.466 | 1.35 | 0.4 | 0.69 | 0.36 | 1.22 | 0.24 |
| IFN-g | 0.85 | 0.677 | 1.04 | 0.125 | 0.85 | 0.68 | 1.04 | 0.13 |
| MMP-9 | 1.14 | 0.86 | 1059 | 0.39 | 1.08 | 0.806 | 1.52 | 0.64 |
| CXCL12 | 2.1 | 1.01 | 5.5 | 0.084 | 1.94 | 0.97 | 4.9 | 0.09 |
| Amphiregulin | 1.78 | 0.91 | 3.76 | 0.105 | 1.44 | 0.675 | 3.24 | 0.353 |
| MMP-7 | 1.21 | 0.63 | 2.35 | 0.57 | 1.14 | 0.58 | 2.25 | 0.69 |
| P-selectin | 1.1 | 0.57 | 2.14 | 0.77 | 0.89 | 0.44 | 1.8 | 0.75 |

**Plasma proteins measured at 24h and their association with fibroproliferation as identified on CT scan.** Odds ratio (OR), 95% Confidence Interval (CI), and *p*-value for association between log_2_-transformed protein concentrations and in-hospital mortality. Logistic model is unadjusted, or includes age, sex, treatment with steroids, and enrollment APACHE III score as covariates. (*) indicates *p*-value<0.05

**Supplemental Table 7**

| **Mediator** | **Unadjusted** | | | | **Age, Sex** | | | |
| --- | --- | --- | --- | --- | --- | --- | --- | --- |
|  | **OR** | **CI (2.5%)** | **CI (97.5%)** | **p-value** | **OR** | **CI (2.5%)** | **CI (97.5%)** | **p-value** |
| CCL2 | 0.61 | 0.0511 | 4.7 | 0.64 | 0.63 | 0.036 | 7.04 | 0.7 |
| MCP-4 | 2.56 | 0.11 | 130 | 0.56 | 4.41 | 0.127 | 505 | 0.442 |
| IL-6 | 0.75 | 0.169 | 1.65 | 0.58 | 0.784 | 0.06 | 2.35 | 0.73 |
| TNF-a | 0.498 | 0.045 | 3 | 0.47 | 0.166 | 0.146 | 34146 | 0.31 |
| IFN-g | 1.03 | 0.47 | 2.65 | 0.94 | NA |  |  |  |
| MMP-9 | 0.92 | 0.57 | 1.46 | 0.7 | 0.914 | 0.57 | 1.46 | 0.71 |
| CXCL12 | 1.01 | 0.578 | 1.84 | 0.96 | 1.01 | 0.57 | 1.84 | 0.98 |
| Amphiregulin | 1.77 | 0.995 | 3.39 | 0.0628 | 1.03 | 0.307 | 3.7 | 0.96 |
| MMP-7 | 1.68 | 1.05 | 2.85 | 0.0379 (*) | 1.72 | 1.02 | 3.06 | 0.0498 (*) |
| P-selectin | 1.14 | 0.49 | 2.72 | 0.76 | 1.137 | 0.48 | 2.75 | 0.77 |

| **Mediator** | **Age, Sex, Steroids** | | | | **Age, Sex, Steroids, APACHE III Score** | | | |
| --- | --- | --- | --- | --- | --- | --- | --- | --- |
|  | **OR** | **CI (2.5%)** | **CI (97.5%)** | **p-value** | **OR** | **CI (2.5%)** | **CI (97.5%)** | **p-value** |
| CCL2 | NA |  |  |  | NA |  |  |  |
| MCP-4 | NA |  |  |  | NA |  |  |  |
| IL-6 | NA |  |  |  | NA |  |  |  |
| TNF-a | NA |  |  |  | NA |  |  |  |
| IFN-g | NA |  |  |  | NA |  |  |  |
| MMP-9 | 0.86 | 0.51 | 1.42 | 0.55 | 0.74 | 0.43 | 1.26 | 0.28 |
| CXCL12 | 1.06 | 0.58 | 1.98 | 0.85 | 0.95 | 0.5 | 1.84 | 0.88 |
| Amphiregulin | 2.3 | 1.18 | 5.1 | 0.0228 (*) | 2.15 | 0.11 | 4.83 | 0.038 (*) |
| MMP-7 | 1.6 | 0.96 | 2.79 | 0.081 | 1.44 | 0.84 | 2.6 | 0.195 |
| P-selectin | 1.18 | 0.47 | 3.05 | 0.72 | 1.02 | 0.4 | 2.7 | 0.96 |

**Plasma proteins measured at 72-168h and their association with fibroproliferation as identified on CT scan.** Odds ratio (OR), 95% Confidence Interval (CI), and *p*-value for association between log_2_-transformed protein concentrations and in-hospital mortality. Logistic model is unadjusted, or includes age, sex, treatment with steroids, and enrollment APACHE III score as covariates. NA indicates too much missingness in the data to correctly calculate the regression model. (*) indicates *p*-value<0.05

**Supplemental Table 8**

| **Mediator** | **Unadjusted** | | | |
| --- | --- | --- | --- | --- |
|  | **OR** | **CI (2.5%)** | **CI (97.5%)** | **p-value** |
| CCL2 | 2.07 | 0.3 | 31.8 | 0.49 |
| MCP-4 | 0.008 | 1.10E-08 | 6.9 | 0.312 |
| IL-6 | 2.03 | 0.53 | 12.67 | 0.33 |
| TNF-a | 1.72 | 0.066 | 61.7 | 0.7 |
| IFN-g | 0.73 | 0.16 | 1.6 | 0.53 |
| MMP-9 | 0.73 | 0.49 | 1.01 | 0.086 |
| CXCL12 | 0.87 | 0.48 | 1.48 | 0.6 |
| Amphiregulin | 1.148 | 0.61 | 2.24 | 0.67 |
| MMP-7 | 2.14 | 1.12 | 4.46 | 0.028 |
| P-selectin | 0.917 | 0.48 | 1.73 | 0.78 |

**Change in plasma protein concentration over time and association with development of fibroproliferation as identified on CT scan.** Odds ratio (OR), 95% Confidence Interval (CI), and *p*-value for association between log_2_-transformed protein concentrations and in-hospital mortality. Logistic model is unadjusted. (*) indicates *p*-value<0.05

**Supplemental Table 9**

| **Immunosuppression** | **Cohort**  (*n*=195) | **Patients with CT scans**  (*n*=75) | | |
| --- | --- | --- | --- | --- |
|  |  | Fibrosis (*n*=20) | No Fibrosis (*n*=41) | *p*-value |
| Dexamethasone, *n* (%) | 147 (75 %) | 18 (90 %) | 35 (85 %) | 0.62 |
| Tocilizumab, *n* (%) | 8 (4 %) | 0 | 1 (2.4 %) | 0.48 |
| Remdesivir, *n* (%) | 83 (43 %) | 5 (25 %) | 23 (56 %) | 0.02 (*) |
| Hydroxychloroquine, *n* (%) | 12 (6 %) | 1 (5 %) | 2 (5 %) | 0.98 |
| Convalescent Plasma, *n* (%) | 23 (12 %) | 4 (20 %) | 5 (12 %) | 0.42 |

**Treatment of COVID-19 with immunosuppression varied among cohort.** *p* -value reflects the difference in proportion of treated compared to untreated among patients with CT scans using a two-tailed z-test.

**Supplemental Figures:**

**Figure S1**


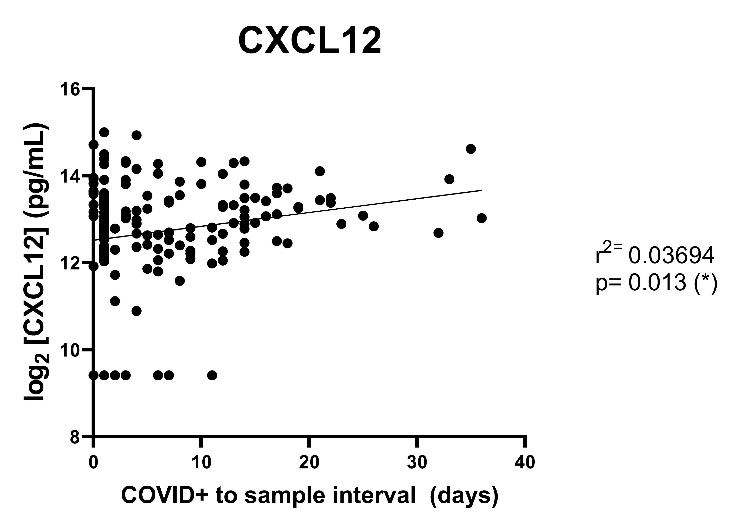

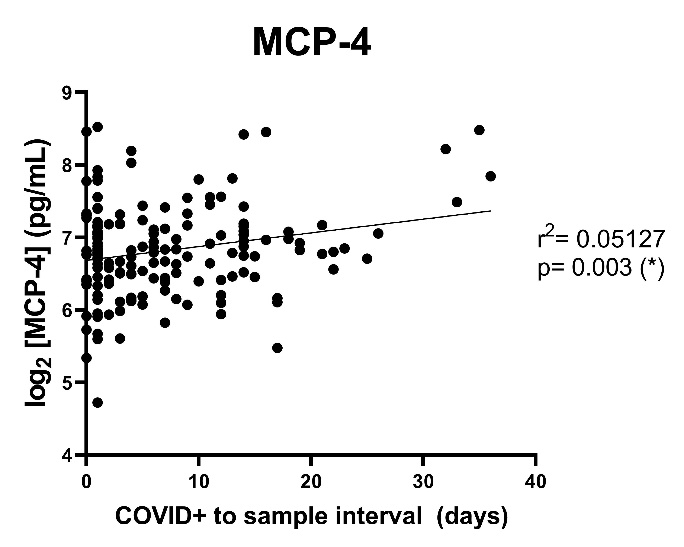


**Supplemental Figure 1.** Association between time interval of COVID+ test to sample collection and log_2_-transformed protein concentration. *p-*value reflects simple linear regression determining whether slope is significantly non-zero.

**Figure S2**
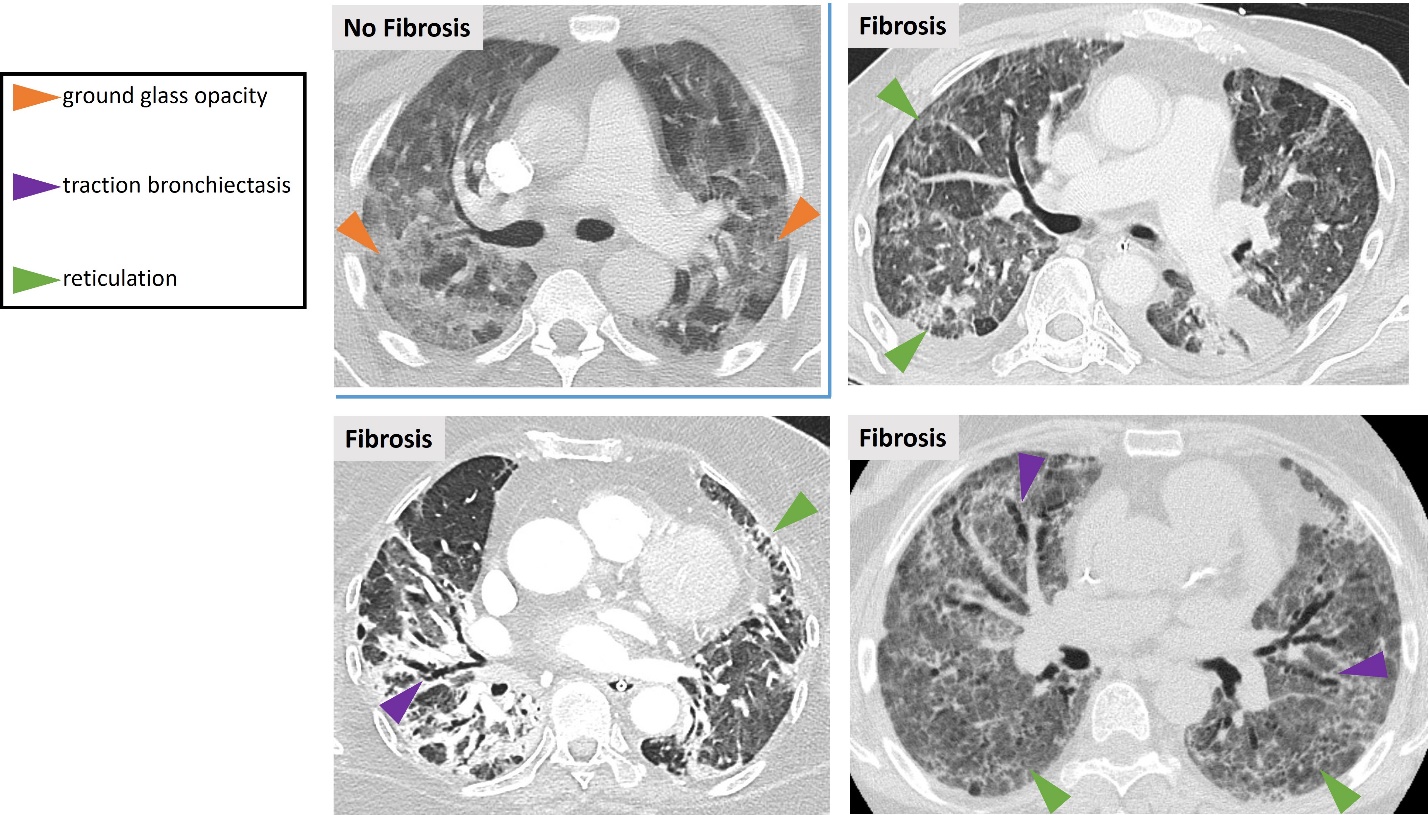


**Supplemental Figure 2:** Examples of CT scans with features of inflammation (ground glass opacity) and fibroproliferation (reticulation, traction bronchiectasis)

**Figure S3**

**(a)**


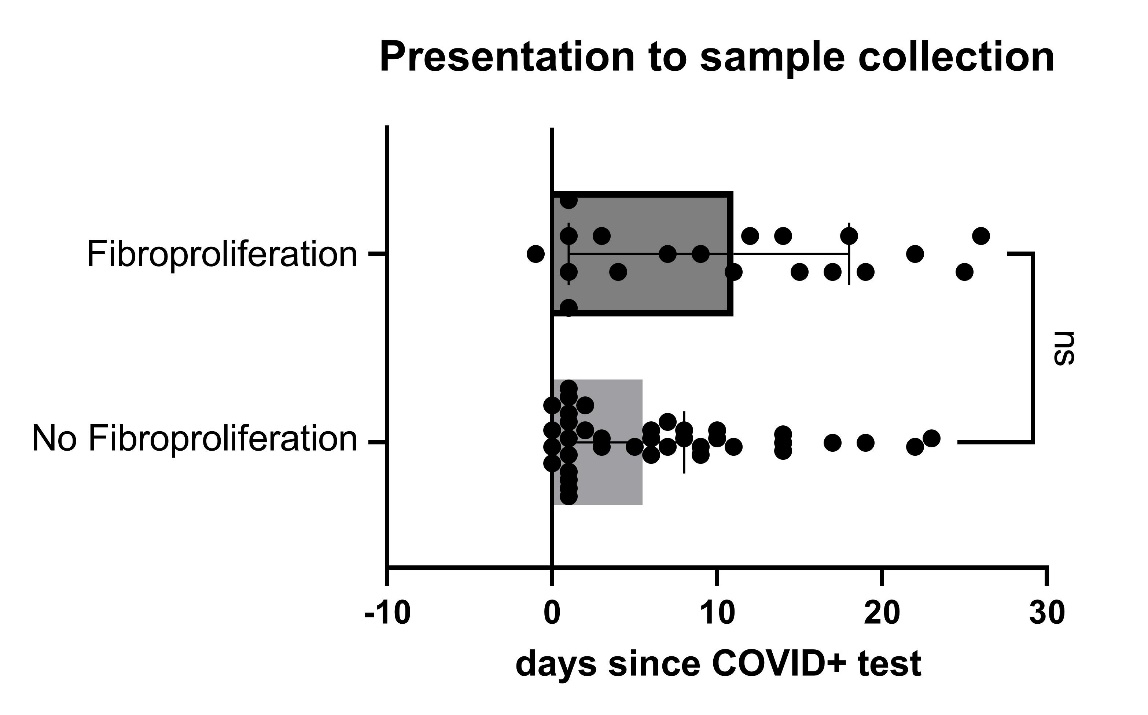


**(b)**


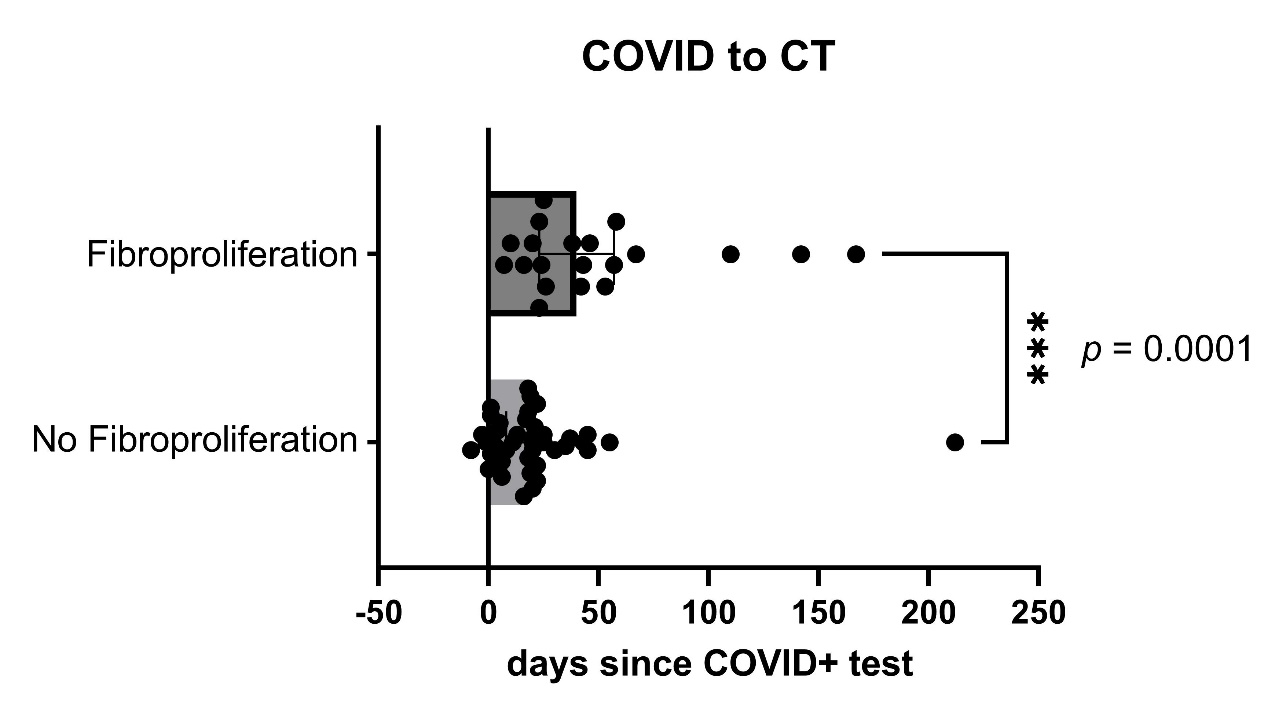


**Supplemental Figure 3:** Intervals of time between COVID+ test and (a) first sample collection and (b) CT scan used to determine fibroproliferation. *p* values reflect two-tailed Mann-Whitney tests of distribution

**Figure S4**


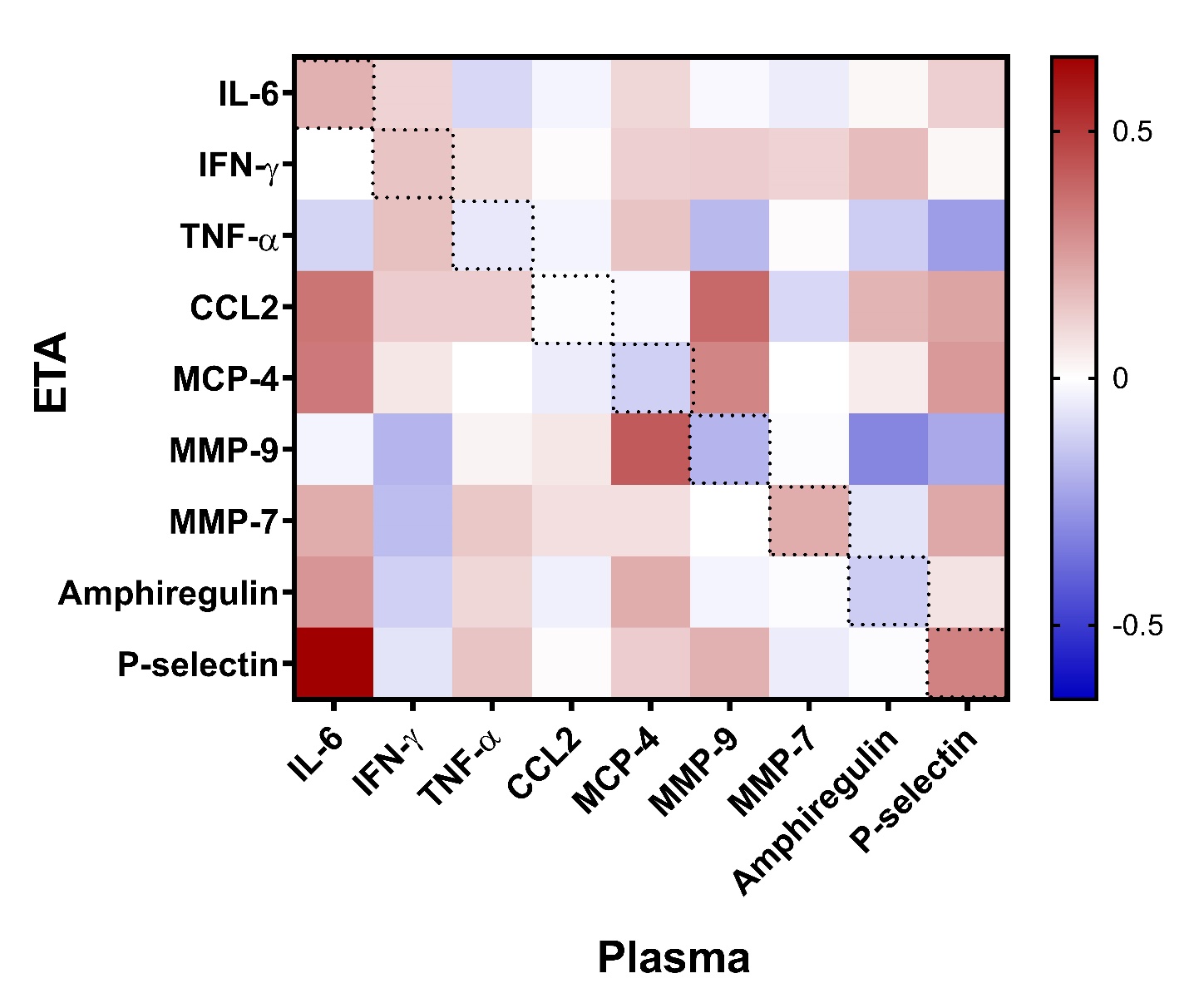


**Supplemental Figure 4:** Biomarker measurements are not correlated between plasma and endotracheal aspirates (ETA) for individual markers. Spearman correlation was used to generate values. Outlined boxes represent the intersection of paired marker tests.
