# Supplementary material for: Mediators of monocyte chemotaxis and matrix remodeling are associated with mortality and pulmonary fibroproliferation in patients with severe COVID-19
Source: PLoS One. 2024 Aug 6;19(8):e0285638. doi: 10.1371/journal.pone.0285638 (PMC11302896; doi:10.1371/journal.pone.0285638)
Supplement: Attachment — Submitted filename: Response_to_Reviewers_Revision2_final.docx [file pone.0285638.s003.docx]

April 26, 2024

Dear Dr. Zissel,

Thank you for your comments and suggestions regarding our manuscript titled, “Mediators of monocyte chemotaxis and matrix remodeling are associated with mortality and pulmonary fibroproliferation in patients with severe COVID-19”. We found the reviewers’ comments to be constructive and we have clarified our statistical analyses and methods which are detailed in our response letter. I have provided a detailed point-by-point response of how we addressed all of the concerns the reviewers have made.

Thank you again and best regards,

Sarah Holton, MD, PhD

Acting Instructor

Division of Pulmonary, Critical Care, and Sleep Medicine

University of Washington

***Reviewer 2 General Comments***: *I thank the authors for addressing the review comments rigorously. I am happy to recommend publication of this manuscript.*

***Response to Reviewer 2:*** Thank you for taking the time to extensively review our manuscript and provide your comments, which we feel improved our manuscript.

***Reviewer 3 General Comments:*** *The manuscript describes the analysis of a COVID cohort subjected to ICU with regard to various proteins in plasma and endotracheal aspirates and specifically addresses their significance as biomarkers for the course of the infection and the development of fibrosis.
This work examines an important topic. However, important information is missing that needs to be added to make the study comprehensible.*

**Response to Reviewer 3:** Thank you for thoroughly reviewing our manuscript. We have responded to your comments and made edits to our manuscript/statistical approaches as described below.

***Reviewer 3 Comment 1:*** *the methods section lacks a precise description of how the plasma was purified after blood collection (in EDTA or nitrate tubes). Please add*

**Response to Comment 1:** The blood was collected in cell preparation tubes (CPT) containing buffered sodium citrate and a density gradient solution for the isolation of plasma and mononuclear cells simultaneously. The tubes containing whole blood were centrifuged at 1500g for 20 minutes, at which point the tube contained layers of plasma, mononuclear cells and platelets, followed by granulocytes and erythrocytes separated by a gel layer. The plasma was then aliquoted in 0.5 mL aliquots and frozen down, carefully avoiding the mononuclear cell layer. The aliquots were then frozen down and stored at -80C until further aliquoting/analysis. These methods have been added to our methods section in the text (lines 283-289).

***Reviewer 3 Comment 2:*** *why were the plasma samples thawed twice before analysis? Please add. Normally plasma is aliquoted and used without thawing for protein analysis, as proteins may be degraded due to multiple thawing cycles.*

**Response to Comment 2:** We recognize that the proteins may be degraded during multiple freeze-thaw cycles, which is why we ensured that the plasma underwent the same number of freeze-thaw cycles prior to analysis. Plasma was processed from whole blood on the same day of sample collection and frozen down (1 freeze). 0.5 mL aliquots were subsequently sub-aliquoted onto plates for our multiplexed analysis (1 thaw followed by 1 freeze). Then, the samples were thawed on the day of analysis (1 thaw), in total resulting in 2 freeze-thaw cycles. This process allowed us to measure several hundred samples simultaneously while minimizing assay batch effects.

***Reviewer 3 Comment 3:*** *the statistical analysis is not comprehensible to me. As I understand it, the aim was to analyze whether there is a difference in the amount of cytokines in the plasma of different proteins in different groups of the cohort. For example, the amount of CCL2 in patients who survived or died was compared. Why was a logistic regression analysis carried out here and not multiple t-tests with a correction for false discovery rate?*

**Response to Comment 3:** Our hypothesis was that there would be differences in the amount of cytokines detected in patients who 1) had higher overall in-hospital mortality, 2) fewer ventilator free days, or 3) development of radiographic fibroproliferation. Our pre-determined covariates that could affect cytokine production were age, sex, APACHE III score at the time of enrollment, and treatment with dexamethasone. We used a logistic regression analysis in order to address/control these covariates.

To demonstrate that the statistical approach does not change our conclusions, please see below a table that shows the p-values based on Mann-Whitney U test for each of our comparisons with the Benjamini-Hochberg correction for false discovery rate. The significantly different cytokines are the same whether the Mann-Whitney test is used, or an unadjusted logistic regression model is used (Supplemental Tables 2-7) A Mann-Whitney test was used rather than a t-test because the cytokine concentrations are not normally distributed.

| **Mediator** | **Mortality – 24h** | | **Mortality – 72h** | |
| --- | --- | --- | --- | --- |
|  | **p-value** | **p-value (fdr)** | **p-value** | **p-value (fdr)** |
| CCL2 | 0.008 | 0.01 (*) | 0.03 | 0.04 (*) |
| MCP-4 | 0.48 | >0.99 | 0.11 | 0.22 |
| IL-6 | 0.0008 | 0.0009 (*) | 0.06 | 0.1 |
| TNF-a | <0.0001 | <0.0001 (*) | 0.023 | 0.02 (*) |
| IFN-g | 0.72 | >0.99 | 0.03 | 0.04 (*) |
| MMP-9 | 0.07 | 0.14 | 0.35 | >0.99 |
| CXCL12 | 0.0025 | 0.003 (*) | 0.5 | >0.99 |
| Amphiregulin | 0.0002 | 0.0002 (*) | 0.0001 | <0.0001 (*) |
| MMP-7 | 0.12 | 0.3 | 0.89 | >0.99 |
| P-selectin | 0.22 | 0.7 | 0.19 | 0.5 |

| **Mediator** | **VFDs – 24h** | | **VFDs – 72h** | |
| --- | --- | --- | --- | --- |
|  | **p-value** | **p-value (fdr)** | **p-value** | **p-value (fdr)** |
| CCL2 | <0.0001 | 0.0002 (*) | 0.048 | 0.12 |
| MCP-4 | 0.2 | >0.99 | 0.76 | >0.099 |
| IL-6 | <0.0001 | 0.0002 (*) | 0.007 | 0.008 (*) |
| TNF-a | <0.0001 | 0.0002 (*) | 0.028 | 0.046 (*) |
| IFN-g | 0.97 | >0.99 | 0.13 | 0.43 |
| MMP-9 | <0.0001 | 0.0002 (*) | 0.45 | >0.099 |
| CXCL12 | <0.0001 | 0.0002 (*) | 0.0016 | 0.001 (*) |
| Amphiregulin | <0.0001 | 0.0002 (*) | 0.02 | 0.025 (*) |
| MMP-7 | 0.0001 | 0.0002 (*) | 0.003 | 0.003 (*) |
| P-selectin | <0.0001 | 0.0002 (*) | 0.025 | 0.036 (*) |
|  |  | |  |  |
| **Mediator** | **Fibroproliferation – 24h** | | **Fibroproliferation – 72h** | |
|  | **p-value** | **p-value (fdr)** | **p-value** | **p-value (fdr)** |
| CCL2 | 0.85 | >0.99 | NA | NA |
| MCP-4 | 0.88 | >0.99 | NA | NA |
| IL-6 | 0.6 | >0.99 | NA | NA |
| TNF-a | 0.71 | >0.99 | NA | NA |
| IFN-g | 0.4 | 0.4 | NA | NA |
| MMP-9 | 0.42 | 0.5 | 0.57 | >0.99 |
| CXCL12 | 0.22 | 0.2 | 0.55 | >0.99 |
| Amphiregulin | 0.31 | 0.3 | 0.04 | 0.08 |
| MMP-7 | 0.68 | >0.99 | 0.05 | 0.1 |
| P-selectin | 0.6 | >0.99 | 0.86 | >0.99 |

***Reviewer 3 Comment 4:*** *A detailed description for how the adjustments regarding age, sex, treatment with dexamethasone, and enrollment APACHE III score were performed, is missing. Please add.*

**Response to Comment 4:** We performed the logistic regression analysis in R studio. Adjustments regarding age, sex, treatment with dexamethasone, and enrollment APACHE III score were performed by including these as covariates in our model. We used the glm function in R with the “binomial” family to specify a logistic regression where the dependent variable was the binary variable of death, fibrosis, or VFDs (high vs low).

This is also described in the methods section, lines 323-326.

***Reviewer 3 Comment 5:*** *Please add the detection limit of the proteins in the electrochemiluminescent immunoassays. Were they around 0.001 pg/ml? Otherwise a negative value of log2=-10 is not possible (TFN-a Figure2).*

**Response to Comment 5:** The lower limit of detection for TNF-a was 0.028 pg/mL. The data point you are referring to was below the lower limit of detection and we have imputed a value of 0.014, resulting in the datapoint shown. This has been clarified in the text (Lines 298-300). We have added the limits of detection for each analyte to Supplemental Table 1, which is copied below:

| **Biomarker Quality Control - Plasma** | | | | | | |
| --- | --- | --- | --- | --- | --- | --- |
| **Biomarker** | **Lower limit of detection (avg, pg/mL)** | **Upper limit of detection (pg/mL)** | **Out of range/imputed (%)** | **Average Intraplate % CV** | **Interplate %CV** | **Included in analysis** |
| IL-6 | .098 | 767 | 1 | 8.71 | 4.65 | Yes |
| IFN-γ | .11 | 1380 | 6 | 8.1 | 4.8 | Yes |
| TNF-α | .028 | 390 | 1 | 5.9 | 4.9 | Yes |
| CCL-2/MCP-1 | 0.02 | 544 | 0 | 5.4 | 10.4 | Yes |
| CCL-13/MCP-4 | 1.72 | 683 | 0 | 6 | 18.9 | Yes |
| Amphiregulin | 1.1 | 2600 | 0 | 7.6 | 23.8 | Yes |
| MMP-9 | 2.3 | 100000 | 0.3 | 4.8 | 15.2 | Yes |
| MMP-7 | 0.67 | 1000 | 0 | 4 | 7.5 | Yes |
| P-Selectin | 5.1 | 200000 | 0 | 3.4 | 7.2 | Yes |
| S100A12 | 15.7 | 50000 | 26 | 10.3 | 26.2 | No |
| SDF-1a/CXCL12 | 3432 | 1020000 | 7.7 | 11.3 | 22.7 | Yes |
| TGF-β1 | 144 | 178500 | 2.5 | 22 | 37.3 | No |

| **Biomarker Quality Control – Endotracheal Aspirate (ETA)** | | | | | | |
| --- | --- | --- | --- | --- | --- | --- |
| **Biomarker** | **Lower limit of detection (avg, pg/mL)** | **Upper limit of detection (pg/mL)** | **Out of range/imputed (%)** | **Average Intraplate % CV** | **Interplate %CV** | **Included in analysis** |
| IL-6 | .04 | 767 | 0 | 20.8 | 48.7 | No |
| IFN-γ | .11 | 138 | 67 | 1.5 | 12.1 | No |
| TNF-α | .03 | 390 | 3.5 | 1.4 | 3.5 | Yes |
| CCL-2/MCP-1 | .06 | 544 | 2.5 | 3.2 | 14.9 | Yes |
| CCL-13/MCP-4 | 1.17 | 683 | 33 | 9.3 | 68.9 | No |
| Amphiregulin | 0.46 | 2600 | 0 | NA | 6.7 | Yes |
| MMP-9 | 1.03 | 100000 | 0 | NA | 4.8 | Yes |
| MMP-7 | 0.95 | 10000 | 3.5 | 2.2 | 5.9 | Yes |
| P-Selectin | 1.8 | 200000 | 1.4 | NA | 12.5 | Yes |
| S100A12 | 50000 | 13.4 | 9.7 | 6.7 | 7.1 | Yes |
| SDF-1a/CXCL12 | 934 | 620000 | 52.6 | NA | 44 | No |
| TGF-β1 | 178500 | 748 | 68.4 | NA | NA | No |

***Reviewer 3 Comment 6:*** *line 353: "Figure 4" is missing*
**Response to Comment 6:** Thank you for catching this error, we have added this in the text (line 365 )

***Reviewer 3 Comment 7:*** *Figure S4: CRP, MCP-4 and CXCL12 are missing, please add and remove CCL-13, as this protein is not shown in another figure/table*
**Response to Comment 7:** Thank you for this point. MCP-4 is another name for CCL13. The graph has been adjusted to reflect that label. CXCL12 was not included as the CXCL12 endotracheal aspirate test characteristics did not meet our quality control standards. We did not discuss CRP in the manuscript and it has been removed from all of the tables.

**Supplemental Figure 4:** Biomarker measurements are not correlated between plasma and endotracheal aspirates (ETA) for individual markers. Spearman correlation was used to generate values. Outlined boxes represent the intersection of paired marker tests.
